# Poultry Meat and Eggs as an Alternative Source of n-3 Long-Chain Polyunsaturated Fatty Acids for Human Nutrition

**DOI:** 10.3390/nu14091969

**Published:** 2022-05-08

**Authors:** Alice Cartoni Mancinelli, Simona Mattioli, Cornelia Twining, Alessandro Dal Bosco, Ann M. Donoghue, Komala Arsi, Elisa Angelucci, Diletta Chiattelli, Cesare Castellini

**Affiliations:** 1 Department of Agricultural, Food and Environmental Science, University of Perugia, Borgo XX Giugno, 74, 06100 Perugia, Italy; simona.mattioli@unipg.it (S.M.); alessandro.dalbosco@unipg.it (A.D.B.); elisa.angelucci@unipg.it (E.A.); diletta.chiattelli@libero.it (D.C.); cesare.castellini@unipg.it (C.C.); 2 Department of Fish Ecology and Evolution, Eawag: Swiss Federal Institute of Aquatic and Technical Sciences, Seestrasse 79, 6047 Kastanienbaum, Switzerland; cornelia.twining@gmail.com; 3 Poultry Production and Product Safety Research Unit, ARS, USDA, Fayetteville, AR 72701, USA; annie.donoghue@usda.gov (A.M.D.); karsi@uark.edu (K.A.)

**Keywords:** poultry genotype, long-chain polyunsaturated fatty acids, antioxidants

## Abstract

The beneficial effects of n-3 long-chain polyunsaturated fatty acids (n-3 LC-PUFA) on human health are widely known. Humans are rather inefficient in synthesizing n-3 LC-PUFA; thus, these compounds should be supplemented in the diet. However, most Western human diets have unbalanced n-6/n-3 ratios resulting from eating habits and the fact that fish sources (rich in n-3 LC-PUFA) are not sufficient (worldwide deficit ~347,956 t/y) to meet the world requirements. In this context, it is necessary to find new and sustainable sources of n-3 LC-PUFA. Poultry products can provide humans n-3 LC-PUFA due to physiological characteristics and the wide consumption of meat and eggs. The present work aims to provide a general overview of the main strategies that should be adopted during rearing and postproduction to enrich and preserve n-3 LC-PUFA in poultry products. The strategies include dietary supplementation of α-Linolenic acid (ALA) or n-3 LC-PUFA, or enhancing n-3 LC-PUFA by improving the LA (Linoleic acid)/ALA ratio and antioxidant concentrations. Moreover, factors such as genotype, rearing system, transport, and cooking processes can impact the n-3 LC-PUFA in poultry products. The use of a multifactorial view in the entire production chain allows the relevant enrichment and preservation of n-3 LC-PUFA in poultry products.

## 1. Introduction

The beneficial functions of long-chain polyunsaturated fatty acids of the n-3 series (n-3 LC-PUFA) in human health are well-known, as well as the importance of having a lower n-6/n-3 fatty acids ratio in diets [1]. However, humans are rather inefficient in synthesizing LC-PUFA; consequently, a certain amount of these compounds need to be acquired directly from the diet [2]. The current eating patterns, mainly in Western countries, result in an excessive intake of n-6 with a consequent n-6/n-3 unbalanced ratio [3]. This has led to a need to increase the n-3 content of foods. However, the benefits of an increase in n-3 LC-PUFA consumption for human health are in contrast with the difficulty in finding sustainable sources of these fatty acids. Fish are one of the richest sources of n-3 LC-PUFA, but, due to the potential of low sustainability of both fishing and aquaculture, it is important to shift the focus to an n-3 source derived from terrestrial animals with higher nutritional value and possibly with lower environmental impacts.

It is well-known that, by altering the composition of animal feed, it is possible to modify the fatty acid profiles of products (e.g., milk, egg, meat [4]). It is important to stress that, due to the hydrogenated activity of microorganisms of the rumen in polygastric animals, there are closer relationships between the fatty acid composition of feeds and animal products in monogastric species. For example, it has been reported [5] that rabbits fed a diet enriched with n-3 precursor (C18:3n-3, α-Linoleic acid, ALA) exhibited meat with higher levels of n-3 LC-PUFA. Progressive increases in the n-3 content of eggs have been shown in laying hens fed diets containing 10% and 20% of n-3 precursors [6].

This review aims to provide a general overview of the importance of n-3 LC-PUFA in human diets and focuses on ways to increase their content in terrestrial animal products. Due to its physiological characteristics (monogastric species and short rearing cycle) and the widespread consumption of meat and eggs, poultry is considered to provide suitable terrestrial sources of n-3 LC-PUFA. The various factors related to increasing and preserving n-3 LC-PUFA in chicken products throughout the production chain are also outlined. In particular, the effects of the nutritional strategies, genotype, and the adopted rearing system are discussed. Further, to preserve the obtained n-3 LC-PUFA, the transport of the birds to the slaughterhouse and the cooking processes are also addressed. 

## 2. Evolution of Human Diet

Early records of human diet date back to the Paleolithic period when the development of small utensils made plant resources, such as roots and tubers, more accessible for human consumption [7]. A key event that influenced the evolution of several current human diets was the domestication of animals and the cultivation of plants. Furthermore, the development of technological processes changed the nutrient components related to “wild” foods. Compared with their domesticated relatives, wild animals have consistently low fat content [8]. Most fat in domesticated animals is characterized by high levels of saturated fatty acids (SFA), some of which can negatively impact human health in terms of cardiovascular disease risk (CVD [9,10]), while polyunsaturated fatty acid (PUFA) and mainly n-3 LC-PUFA show numerous positive effects [11], such as preventing pathological disorders (see Section 3.1). For example, the current human diets in the US contain 12% of the total energy as saturated fats [12], while this value in the Paleolithic diet was only 7.5% [13,14]. 

However, the main difference between current versus Paleolithic diets is not in terms of the total fat intake (Paleolithic diet about 35% of total energy intake versus current recommendation of 20–35%) but mainly the ratio between different PUFA [12]. During the early evolutionary history of the genus *Homo*, there was a strict balance between n-6 and n-3 PUFA. In particular, a significant amount of n-3 fatty acids was present in the foods commonly utilized by prehistoric humans, including meat from hunting animals, wild plants, eggs, and fish. The n-3 LC-PUFA are the major structurally significant and biochemically active components of the brain in all mammalian species [15]. The phenomenon described as “encephalization” proposes that the increase in the brain size in primates and humans is probably due to the availability of n-3 LC-PUFA in the diet during early human evolution [16]. However, dietary changes in the last 100 to 150 years have influenced the type and the amount of PUFA, as well as other bioactive molecules (e.g., antioxidant, essential amino acids, micronutrients, etc.) in the food. 

**Figure 1:**
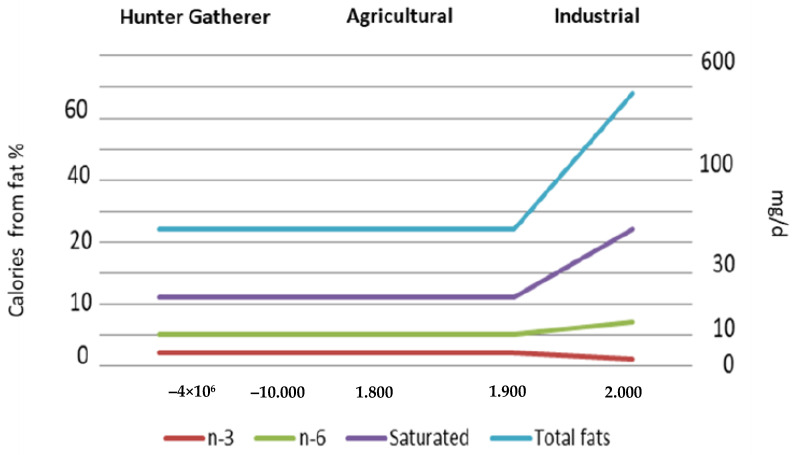
Estimated intake (mg/d) of n-3, n-6, saturated fatty acids, and total fats in relation to calories (%) provided by fat during human evolution. Data from Simopoulos, 2019 [22].

The best available estimate of ancestral human intake showed a different ratio between n-6 and n-3 (about 2:1) [14,17]. In contrast, the estimated PUFA consumption for humans today in the USA is about 15 g/d, but the intake of n-6 LC-PUFA is 10-fold higher than n-3 LC-PUFA [18]. In EU countries, there is a wide variation in LC-PUFA intake [19], with the amount of Eicosapentaenoic (C20:5n-3, EPA) plus Docosahexaenoic (C22:6n-3, DHA) acid varying between 106 and 419 mg/d, which is below the daily recommendations (500 mg/d). In critical population groups (e.g., infants, children, adolescents, elderly, and pregnant/lactating women) the ALA intake is within the daily dose recommendation in 77% of the EU countries, whereas the n-3 LC-PUFA intake met the recommendations only in 26% of the European countries. These results indicate that the intake of n-3 and n-6 PUFA is suboptimal in specific population groups in Europe [20] and that their ratio is about 12 times higher than the recommendations [21] and has increased since 1961. Thus, in Western countries, human diets are unbalanced in terms of PUFA, with a significant increase in n-6 and a decrease in n-3 LC-PUFA. Overall, during the last 100 years, the ratio between n-6 and n-3 has significantly increased to 20:1, whereas the recommendations suggest that this ratio should be less than 4:1 (Figure 1).

## 3. Notes on Metabolism of Essential Fatty Acids and Desaturase Activity (Details of Synthesis)

**Figure 2:**
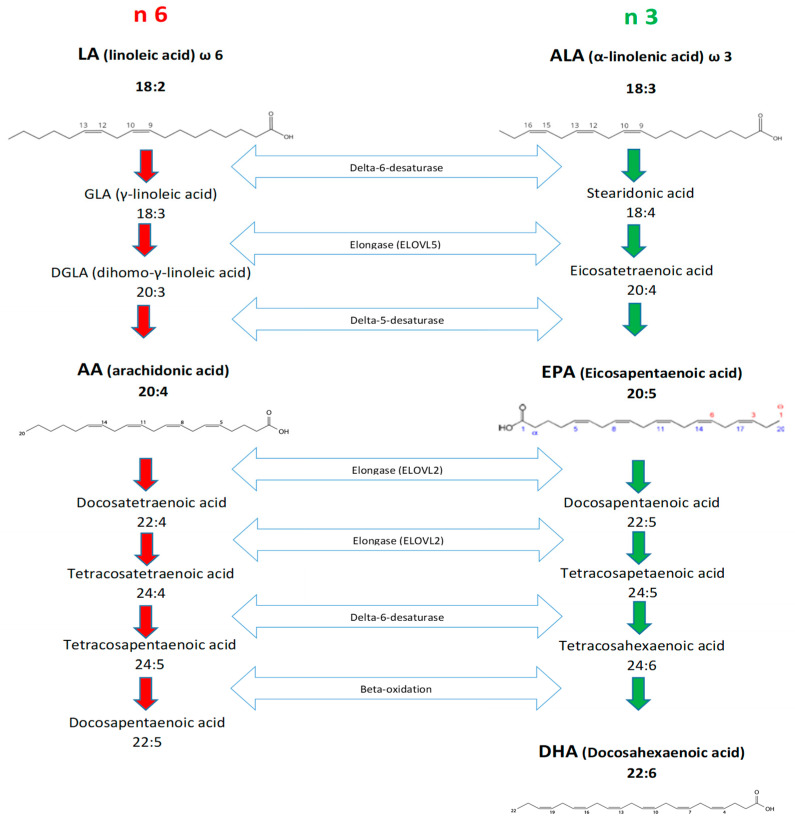
Metabolic pathways of n-6 and n-3 PUFA. The red arrows indicate the synthesis of n-6 and the green arrows the synthesis of n-3. The double blue arrows represent the enzymatic pathway.

Linoleic acid (C18:2n-6, LA) and ALA are the precursors of n-6 and n-3 LC-PUFA, respectively. These precursors cannot be synthesized by mammals, including humans, and birds, and, for this reason, they are defined as essential fatty acids (EFA; [23]). The most physiologically important n-3 LC-PUFA are EPA and DHA, whereas Arachidonic acid (C20:4n-6, AA) is the most important n-6 LC-PUFA [23]. Both LA and ALA can be converted into long-chain metabolites through an elongation and desaturation metabolic process (Figure 2), which predominantly occurs in the liver. Other tissues (e.g., brain, testicles, epididymis, ovaries, muscles), although capable of synthesizing a certain amount of LC-PUFA, have lower metabolic efficiency [24,25,26]. The Δ5- and Δ6-desaturases enzymatic complex, controlled by the fatty acid desaturase 1 and 2 (FADS1 and FADS2) genes, introduces double bonds into the respective fatty acids (i.e., LA or ALA). The FADS2 acts twice, first at the C18 level and second after the conversion of the latter into C24 derivatives; accordingly, it is considered one of the main factors limiting LC-PUFA biosynthesis.

The conversion of ALA into EPA is in competition with the conversion of LA into AA because the biosynthesis of both n-3 and n-6 LC-PUFA requires the same enzymes [23] (Figure 2). This competition for desaturases and elongases in n-3 and n-6 LC-PUFA synthesis affects their relative concentration in tissues. Thus, animals fed diets rich in n-6 produce more LA metabolites, such as AA, compared to ALA metabolites, such as EPA and DHA, whereas higher ALA intake results in increased synthesis of n-3 LC-PUFA [26].

These two PUFA families, ALA and EPA for the n-3 and AA for the n-6 series, are the precursors of the main compounds involved in the inflammatory response, such as prostaglandins (PGs), thromboxanes (TH), and leukotrienes (LT) [27]. Such molecules, called eicosanoids, have opposite functions: anti-inflammatory and anti-aggregating properties when synthesized by n-3 LC-PUFA, whereas pro-inflammatory and aggregating properties when derived by n-6 LC-PUFA. In particular, Cyclooxygenase (COX) is the key enzyme in the synthesis of PGs from AA, and it is present in two isoforms:

1. COX-1, a constitutive enzyme widely expressed in most tissues because it controls the synthesis of PGs involved in the regulation of homeostatic function;
2. COX-2, a specific enzyme, exerts its functions only during inflammatory processes; thus, PGs formed by COX-2 are principally involved as mediators of pain and inflammation [27,28].

**Figure 3:**
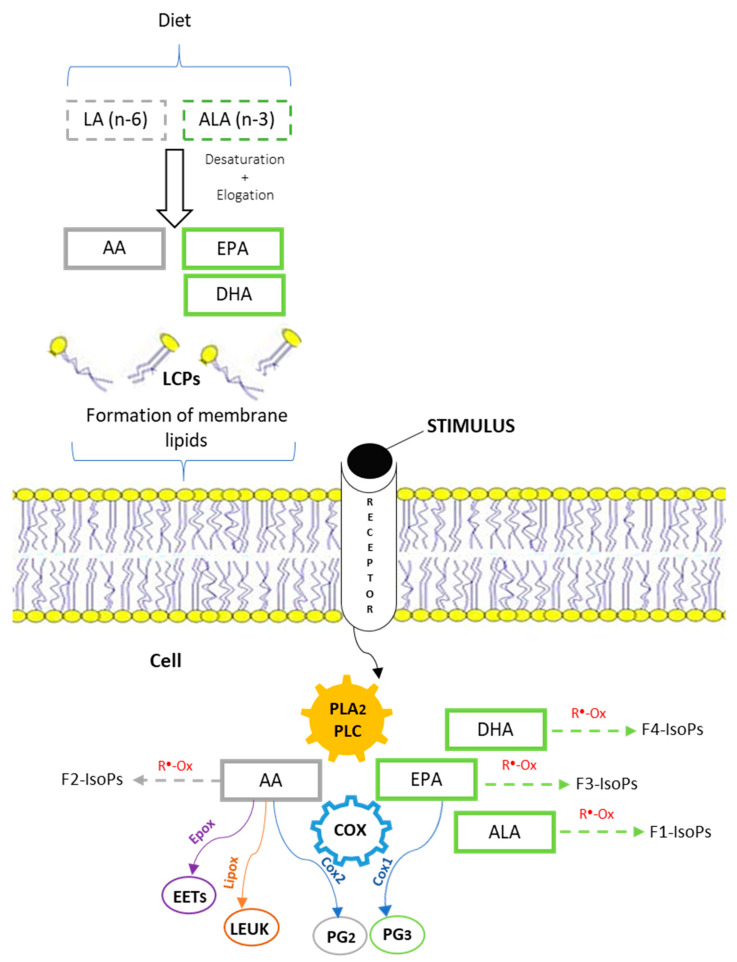
Schematic representation of PUFA metabolism, PGs, and IsoPs synthesis. The n-3 and n-6 precursors (ALA and LA) from the diet through desaturation and elongation processes form LC-PUFA: AA (n-6), EPA, and DHA (n-3). The LC-PUFA are incorporated into the phospholipids of plasma membrane. When external stimuli occur, the PLA_2_ and PLC cleave phospholipids to increase the cell availability of AA, EPA, ALA, and DHA. The AA is processed by the COX_2_ enzyme to form PG_2_, or by epoxygenase and lipoxygenase to form epoxyeicosatrienoic acids (EETs) and leukotrienes (LEUK). The EPA is involved in the PG_3_ synthesis by the COX_1_ enzyme. Free radical peroxidation of LC-PUFA generates different classes of IsoPs. F_2_-IsoPs derive from AA oxidation, while F_1_, F_3_, and F_4_-ISoPs from ALA, EPA, and DHA, respectively.

The biosynthesis of PGs is triggered following the onset of extracellular stimuli. These stimuli, in turn, trigger the activity of phospholipase A2 (PLA2) and phospholipase C (PLC), which cleaves phospholipids from the plasma membrane and increases the availability of fatty acids for the PGs synthesis by the COX enzyme (Figure 3). The ALA and EPA can be processed by COX-1 to generate PGs of the 3-series (PG_3_, less inflammatory), while AA can be processed by COX-2 to generate prostaglandins of the 2-series (PG_2_) or by epoxygenase and lipoxygenase to form epoxyeicosatrienoic acids (EETs), thromboxanes, leukotrienes, and hydroxyeicosatetraenoic acids (di-HETEsM) [29]. As previously reported, the PG_2_ are highly inflammatory and responsible for the pathophysiological process of fever (e.g., prostaglandin E_2_, PGE_2_). The availability of ALA, EPA, or AA for the PGs synthesis is impacted by the composition of the membrane phospholipids, which, in turn, is influenced by the levels of n-6 and n-3 precursors in the diet.

A significant body of literature demonstrates that increased ingestion of n-3 PUFA is associated with a decreased PG_2_ synthesis [3,30]. This is due to the replacement of AA with n-3 PUFA in the phospholipids, which, when released, attenuates the rate of PG_2_ formation (AA-derived). Accordingly, modification of n-3 and n-6 PUFA (especially AA) availability with different strategies (diet, drugs, etc.) strongly affects the immune and inflammatory responses of the body [31]. Thus, the fatty acid composition of tissues can influence their inflammatory responses. This process of inflammation represents a physiological defense mechanism protecting the body from infection and diseases; however, it must be well-regulated in order to maintain homeostasis (inflammation vs. anti-inflammatory). Because n-6 PUFA content is much greater than n-3 PUFA in typical Western diets, controlling dietary AA allows a down-regulation of PG_2_ synthesis and, consequently, anomalous inflammatory responses [32].

In this context, it is important to mention a new class of compounds, recently discovered, called isoprostanoids (IsoPs). The IsoPs are a series of PGs-like compounds produced by the free-radical-catalyzed peroxidation of fatty acids, independent of the COX [33,34]. On the basis of the LC-PUFA involved, IsoPs can be divided into different classes: F_2_-IsoPs derived from AA oxidation; F_1_, F_3_, and F_4_-ISoPs from ALA, EPA, and DHA, respectively [35]. Because of their specific derivation, IsoPs are considered a very sensitive marker of lipid peroxidation. Recent studies [36,37,38] have established that these molecules, besides being robust markers of oxidative damage, also exhibit a wide range of biological activities. F_2_-IsoPs exert a vasoconstriction function, especially at the renal level; the F_4_-ISoPs, also called neuroprostane (4-F_4_t-NeuroP), is mainly present in brain tissue and increases its concentration with the onset of neurodegenerative disease. F_1_-ISoPs, because of its origin from ALA, is considered the main oxidation biomarker in plants, enhancing its level under stress conditions. Due to issues with the imbalance ratio of n-6/n-3 in modern diets, it was recommended to increase the amount of n-3 in the human diet, and, subsequently, there has been an increased interest in the research towards the IsoPs derived from EPA and DHA. In fact, since n-3 LC-PUFA exert beneficial functions for human health, it is possible to hypothesize that their derivative compounds can also be beneficial.

Many studies report that a high dietary level of n-3 PUFA leads to the formation of F_3_-IsoPs and F_4_-IsoPs from non-enzymatic oxidation of EPA and DHA, respectively [26]. In addition, their abundance is also affected by the PUFA composition of different organs; for example, the brain and spermatozoa are particularly rich in n-3 LC-PUFA and also in F_4_-IsoPs [26]. However, despite the importance of the IsoPs at the physiological level, further investigations are needed to better understand the different role and metabolic pathways involving such compounds and their relation with the LC-PUFA precursor.

### 3.1. Relevance of n-3 LC-PUFA in Human Nutrition

In the last few decades, the LC-PUFA compounds have been largely investigated for their nutritional value and for the numerous biological actions and therapeutic functions in different organs. The n-3 LC-PUFA are particularly abundant in the brain, retina, and reproductive cells and play important roles in many metabolic pathways, preventing pathological disorders, such as cardiovascular disease, reproductive dysfunction, chronic inflammatory diseases, depression, and deficiencies in the immune system [39,40].

As previously mentioned, humans are rather inefficient in synthesizing LC-PUFA [41] and, accordingly, they should be consumed directly through the diet. Thus, experts, such as the European Food Safety Authority (EFSA), have established n-3 nutritional recommendations in relation to age, sex, and body condition. It has been suggested that 250 mg/d is the minimum quantity of EPA and DHA required for an adult [42].

For pregnancy and lactation, considering the increased demand of n-3 LC-PUFA, the suggested intake is 350 to 450 mg/d of DHA, while, for young children (<24 months), the adequate intake is 100 mg/d of DHA. DHA is the main LC-PUFA recommended as it is a key component of the membrane lipids of the nervous system and adequate DHA concentration is linked to optimal brain development that occurs in the first 2 years of life [1]. In the fetus and newborn, the supply of DHA depends on the maternal diet since studies [43,44] showed that a high n-3 LC-PUFA intake by pregnant or lactating women can promote mental development in babies. On the contrary, low n-3 LC-PUFA concentration during the fetal period affects the brain volume, reducing its dimensions in childhood [45]. Moreover, in the brain, n-3 LC-PUFA, besides the formation of the plasma membrane, exert other functions, such as an increase in cognitive activity [46] and development of synaptic functionality and plasticity [47]. Indeed, a recognized beneficial effect of n-3 LC-PUFA consumption concerns neurodegenerative disorders, such as Parkinson’s (PD) and Alzheimer’s disease (AD) [48,49]. Accordingly, a lower concentration of DHA was found in the serum of patients affected by AD as compared to healthy people [50], suggesting that n-3 LC-PUFA could represent a preventative strategy against AD, especially when consumed in the early stage of the disease [51]. Numerous clinical trials were carried out to investigate the effects of n-3 consumption on the reduction in CVD; however, an open debate is underway regarding the efficacy. Recent studies support the hypothesis that the intake of n-3 LC-PUFA reduces the risk of CVD [52,53], but not all the research papers agree with this outcome [54] as varying experimental designs and the heterogenicity of the results render it difficult to find clear conclusions [55].

The EFSA [56] has performed numerous studies to determine the tolerable upper intake (UI) of n-3, concluding that a higher intake did not induce any adverse effect on human health; therefore, individuals could safely increase their daily n-3 LC-PUFA consumption.

The n-3 LC-PUFA represent about 30 to 50% of the membrane composition of sperm cells and are fundamental for reproductive activity by regulating the fluidity and the acrosomal responsiveness [57,58]. The high LC-PUFA concentration in the spermatozoa plasma membrane makes it vulnerable to lipid peroxidation [59]. Thus, the presence of oxidative stressors, such as obesity, sexually transmitted disease, alcohol, and tobacco use, could represent possible factors affecting male infertility [60,61].

The Western diet rich in saturated fat and n-6 PUFA induces obesity that is associated with many diseases, such as cancers, behavioral disorders, cardiovascular complications, and insulin resistance. Although these pathologies come from different causes and conditions, their onset is linked to the increase in n-6 PUFA and decrease in n-3 PUFA in the modern diet so that they are defined as diet-related chronic diseases. As previously reported, the overconsumption of n-6 PUFA increases the synthesis of PG_2_, with a pro-inflammatory effect. Moreover, it has been recently discovered that the AA-derived endocannabinoid compounds linking to their brain receptors are able to increase the appetite and food intake, worsening obesity status [62]. The AA is widely present in various cells and tissues so that it can be quickly converted into pro-inflammatory eicosanoids, increasing the chronic disorders [62]. The higher AA concentration with respect to EPA and DHA can lead to oxidative stress in the cell due to the increase in reactive oxygen species production [63]. Oxidative and inflammation status can negatively affect the reproductive function and elevate the risk of CVD, cancer, and other chronic diseases. Therefore, increasing the n-3 intake in humans in order to maintain a balanced n-6/n-3 ratio is essential for regulating body homeostasis.

### 3.2. Global Requirements for EPA and DHA

As previously reported, EFSA [42] provides the recommended minimum daily intake of EPA and DHA for different classes of population. Combining these data with those related to the world population classes [64,65], it is possible to estimate the annual global requirement for n-3 LC-PUFA (Table 1). As reported in Table 1, the annual global requirement for n-3 LC-PUFA (mainly EPA + DHA) to satisfy the need of the whole world population is about 722,960 t/y. The main source of n-3 is represented by fish; however, the supply of n-3 LC-PUFA from fish and fish products is insufficient to meet the world n-3 LC-PUFA demand [66]. Consequently, the estimated n-3 LC-PUFA deficit is about 347,956 t/y. It is noteworthy that such estimates do not take into account the most vulnerable groups of population (i.e., elderly and patients affected by physiological and metabolic disorders); thus, the amount of n-3 LC-PUFA deficit could be even higher. Within this context, it is not clear how to satisfy this enormous n-3 LC-PUFA deficit with the current foods. Accordingly, it is vital to develop alternative strategies for increasing n-3 PUFA and mainly n-3 LC-PUFA availability in common foods.

## 4. Sources of n-3 LC-PUFA and Strategies for Enriching Terrestrial Food

The importance of n-3 LC-PUFA in human health is evident, and they have been extensively described herein; however, the problem of how to increase the n-3 intake in the human diet is still unsolved. In this section, the principal n-3 LC-PUFA sources in the human diet (vegetable oil, fish, and terrestrial animal products) are described and new strategies to increase their availability in the food are discussed.

### 4.1. Vegetable Source

The beneficial effects of n-3 LC-PUFA on human health, and the low presence of these compounds in common foods, promoted the development of foods “enriched” in these fatty acids and the development of alternative terrestrial n-3 LC-PUFA sources.

The main sources of PUFA in human diets are vegetable oils. Many plants are able to synthesize ALA by successive desaturation of oleic acid and LA [67]. This process takes place in the plant leaves, roots, and seeds. Although vascular plants exhibit two distinct pathways for PUFA biosynthesis [68], fatty acid biosynthesis occurs almost exclusively in the plastids [69], and it is catalyzed by fatty acid synthase [70], which permits the synthesis of long-chain MUFA (monounsaturated fatty acids), such as erucic acid (C22:1), but the production of AA, EPA, and DHA is null or very scarce [71]. Most of the elongase enzymes involved in glycerolipid metabolism in plants have relatively broad substrate specificities capable of synthesis of fatty acids with 20 and 22 carbon atoms (C20 and C22 fatty acids [72]). Accordingly, LC-PUFA are only marginally synthesized because plants have a low desaturase activity on fatty acids with over 20 carbon atoms [72,73].

Some fungi, bryophytes (i.e., mosses and liverworts), and some marine and freshwater algae are capable of synthesizing LC-PUFA. However, the mechanisms of how these organisms are able to produce these compounds are not known [74,75,76,77]. In particular, these microorganisms, besides having the same desaturases present in the higher plants, show a higher affinity for the C20 and C22 fatty acids. Moreover, while some vascular plants are rich in fats, including ALA and other PUFA (e.g., flaxseed, canola), even when ALA constitutes a considerable fraction of fatty acids [78,79], the ALA content of most plants is low because they are low in fat [80,81,82].

### 4.2. Fish and Fish Products

Fish are the main source of n-3 LC-PUFA for humans, but the content in EPA and DHA varies by species and by how the fish are raised (i.e., wild or farm-raised, warm or cold water) [83]. For example, Pacific cod has higher EPA and DHA content compared to Atlantic cod (0.235 and 85 g/100g vs. 0.134 and 85 g/100g, respectively) [83].

Farm-raised and wild fish often contain similar amounts of EPA and DHA, but farmed fish are typically fed fish meal [84], which is not sustainable at a global scale because wild fish are used to produce fish meal [85]. In addition, the total SFA and PUFA content in farm-raised fish is higher compared to wild fish due to the higher n-6 concentrations in fish feeds [84]. Importantly, a number of marine fish of high commercial value for human consumption are unable to synthesize n-3 LC-PUFA. In fact, studies on hepatocytes from marine fish have shown very low ALA desaturation rates, without the production of EPA or DHA [86]. As previously mentioned, LC-PUFA synthesis depends on the desaturase and elongase activities. The relative inability of some marine fish to produce EPA and DHA can result from limited activities of either C18 or C20 elongases, as well as from low activity of Δ5 desaturase, which converts 20:4n-3 into EPA [87]. These enzymes appear to vary in their efficiency based upon the availability of PUFA in natural ecosystems [88,89]. Marine fish have large amounts of EPA and DHA in their diets, whereas freshwater fish consume diets that are more variable in n-3 LC-PUFA content. For example, carnivorous marine fish (e.g., tuna) lack functional desaturation and elongation enzymes, likely because they have little selective pressure to maintain synthesis in an environment where it is not strictly required [90]. Accordingly, many marine fish are only “accumulators” of n-3 LC-PUFA produced by lower trophic levels (i.e., marine phytoplankton). For example, phytoplankton rich in EPA and DHA include *Bacillariophyceae, and Chrysophyceae; Cryptophyceae, Prasinophyceae, Rhodophyceae, Xanthophyceae, Glaucophyceae and Eustigmatophyceae* (EPA sources), *Dinophyceae, Prymnesiophyceae*, and *Euglenophyceae* (DHA sources) [91,92]. These algae can also have high lipid contents, as well as high n-3 LC-PUFA concentrations (around 30–70% of DHA).

Although all fish originally evolved from marine lineages, in less n-3 LC-PUFA-rich freshwater environments, fish evolved an increased ability to synthesize n-3, including multiple species and populations with relatively recent marine origins [93].

Even though many studies have been carried out to increase the sustainability of aquaculture and, in particular, for the replacement of fish meal with other sources [94,95], fish are becoming progressively scarce and the over-exploitation of fishing areas worldwide is unsustainable. In addition, aquaculture cannot be considered a very sustainable source because, paradoxically, the feed used contains large quantities of wild fish [96]. Beyond the problems of sustainability, there is a growing concern with the methylmercury and polychlorinated biphenyls (PCB) levels in some species of fish, such as swordfish (*Xiphias gladius*), mackerel (including different species of pelagic fish, mostly from the family Scombridae family), and shark (*Selachimorpha*). Hites et al. [97] reported that levels of mercury and PCBs are higher in farm-raised salmon compared to wild salmon. The risks of methylmercury and PCB exposure are even more common in fish fed fish meal [98]. For this reason, the US Environmental Protection Agency [99], the US National Academy of Sciences [100], and additional international medical institutions recommend limiting the consumption of some species of fish.

Therefore, the choice of terrestrial animals as sources of n-3 LC-PUFA, and, thus, their ability to elongate and desaturate PUFA, must be carefully considered in order to find other sustainable, healthy, and safe products.

### 4.3. Terrestrial (Farmed) Animals

Terrestrial animals generally have lower content of n-3 LC-PUFA in their body than fish and a modest ability to synthesize LC-PUFA. Because the development of an embryo and fetus largely depends on LC-PUFA availability [101], females have higher n-3 LC-PUFA content and synthesis capacity than males [102]. Accordingly, female rats showed a higher DHA anabolism than males [102] and, thus, a higher liver concentration of DHA [103].

The most direct relation between the fatty acid profile of feed and that obtained in the food produced by it (meat, egg) is obtained in monogastric species. On the contrary, in ruminants, ingested fatty acids are hydrogenated by microorganisms of the rumen [104]. Stearic acid (C18:0) is the end product of this reaction, and it passes from the rumen into the abomasum, where it is digested and absorbed [105]. Fatty acids from the rumen are precursors of plasma synthesis of triglycerides that are mainly incorporated into the lipids of milk and adipose tissue [104]. Because of the hydrogenation activity of bacteria, the triglycerides of ruminant plasma, milk, and body fat result in a low PUFA content [106]. In ruminants, in order to bypass the rumen, PUFA must be protected for absorption in the duodenum [107]. In this way, the concentration of n-3 PUFA in milk can be increased fourfold by the inclusion of rumen-protected tuna oil in the diet of cows [108].

Unlike ruminants, in monogastric species (poultry, pig, rabbit), the PUFA profile of feed results in corresponding levels in their products; therefore, several strategies for enriching their content can be used. Thanks to these dietary strategies, there are a number of animal products in the market that are enriched in n-3 fatty acids, such as [109]:

1. Meat and poultry products (sausages, frankfurters, etc.);
2. Eggs and egg products (mayonnaise, etc.);
3. Milk and milk products (yoghurt, cheese, etc.).

It should be noted that the current meat and egg supply from monogastric animals is estimated to produce around 75.632 t/n-3 LC-PUFA/year (Table 2), which represents about 21 to 22% of the LC-PUFA annual world requirements, whereas other foods (beef, lamb meat) can add only a minor amount of n-3 LC-PUFA (<1%) [110,111]. It is likely that the major variation in lipid intake between populations reflects the true underlying differences in intakes and types of lipids consumed. Since it is difficult to change dietary habits, it is very important to modify the lipid profile of foods.

In this context, poultry is particularly interesting for the following reasons:

- monogastric animal;
- short breeding cycle; meat-type chickens have an age at slaughtering of about 40 days;
- there are no religious limitations for poultry meat (or not as many as for pork or beef/lamb);
- lower environmental impact than other livestock productive chain [112] due to the high efficiency in converting feed into food;
- it is the most-consumed meat in the world;
- eggs easily meet the EFSA recommendation for n-3-enriched foods.

#### Poultry Meat and Eggs as Functional Foods

Poultry meat is rich in protein and is very suitable for human nutrition due to its low-fat content, high unsaturation degree of fatty acid, and low cholesterol levels [113]. It can even be considered to be a “*functional food*” as poultry meat can be beneficial for human health because it contains bioactive substances, such as vitamins and antioxidants, and has a balance of n-6 to n-3 ratio [114] close to the recommended ratio of 4:1.

It is also well-established that poultry eggs contain vitamins and minerals in addition to biologically active compounds with antimicrobial, immunomodulator, antioxidant, anti-cancer, or anti-hypertensive properties [115]. Owing to their high nutritional value and positive effects on human health, several of these compounds found in eggs are also selectively isolated and produced on an industrial scale [116]. Numerous studies have demonstrated that the quality of poultry meat and eggs can be further improved through various methods, including manipulating the diet to target certain functionalities, leading to the concept of the chicken as a bioreactor for the production of substances for humans [117,118,119].

## 5. n-3 LC-PUFA in Poultry Meat and Eggs, Strategies of Enrichment

Several studies have demonstrated that it is possible to increase the n-3 LC-PUFA content of animal origin products through different strategies, such as dietary supplementation, genetic selection, and rearing systems management. Moreover, pre- (transport to slaughterhouse) and post- (cooking) mortem factors can affect the preservation of the n-3 LC-PUFA enrichment in foods (see Section 8 and Section 9).

### 5.1. Dietary Strategies for Broilers and Laying Hens

**Figure 4:**
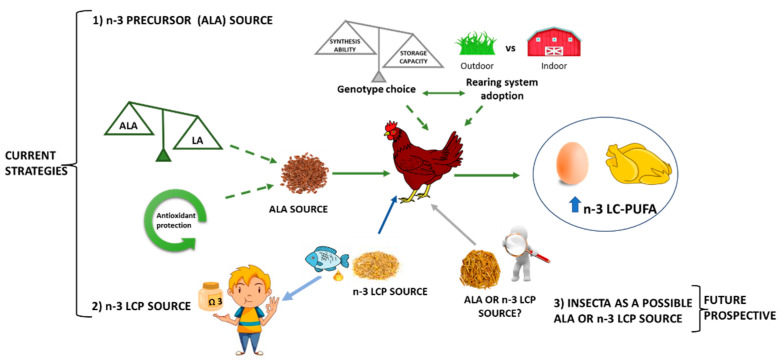
Graphical representation of the main dietary strategies to increase n-3 LC-PUFA in poultry products (egg and meat). The current dietary strategies consist of: (1) providing to animals n-3 precursor (ALA source) or (2) enriching animal feed with n-3 LC-PUFA. (3) A future perspective could be represented by insects as a source of n-3 precursors or n-3 LC-PUFA.

The n-3 LC-PUFA enrichment of livestock products is based on the dietary supplementation of n-3 PUFA precursors (ALA) from terrestrial sources or n-3 LC-PUFA from marine oils (Figure 4).

The first strategy implies that ALA must be converted by animal metabolism into LC-PUFA, while, in the second case, the LC-PUFA is simply absorbed, transferred, and stored in different tissues. Although this second strategy (e.g., addition of fish oil) easily enriches food in n-3 LC-PUFA, it is highly dependent upon wild fish production in marine ecosystems. In this view, it is important to consider that fish oil is an economically and environmentally expensive additive and the feed represents the major animal production cost [120]. Moreover, the fish oil approach also has other drawbacks [121,122], demonstrated by the fact that its use in the chicken diet could negatively affect the sensory properties of the meat. Furthermore, it is important to point out that fish oil could be considered a constituent of the human diet [123]. In fact, the use of refined fish oil is much more metabolically efficient if administered directly to humans [124] without passing through the metabolism of livestock animals to produce food. Therefore, following these considerations, it is important to enforce the nutritional strategy based on the use of n-3 precursor. Further prospective could be represented by the introduction of insects to a poultry diet as a source of n-3 precursor or n-3 LC-PUFA. However, research still needs to be conducted to validate this aspect, and clarification in the regulations is needed to better understand how to manage the insects rearing (see Section 5.1.3).

By manipulating the broiler and the laying hen diets, it is possible to improve the conversion efficiency of ALA into n-3 LC-PUFA by exploiting the bird metabolism.

Despite broilers and laying hens exhibiting a different nutritional requirement, in order to efficiently administer ALA dietary supplementation, it is important to consider two main aspects (Figure 4):

1. The n-6/n-3 ratio of the diet. In fact, due the involvement of the same enzymes, the n-3 synthesis is in competition with the synthesis of the n-6 one; thus, the higher LA diet presence could reduce the production of EPA and DHA by favoring the AA synthesis [125];
2. The antioxidant supplementation (vitamin E, vitamin C, Selenium, etc.). Due to their double bonds, PUFA are very susceptible to oxidation, resulting in reduced shelf life of feed as well as meat and eggs. This can lead to a poor acceptance of the feed by the animals but also a poor acceptance of n-3 LC-PUFA-enriched products by the final consumers due to the development of unattractive colors or unpleasant tastes and aromas.

#### 5.1.1. Dietary Enrichment for Broilers

In general, chicken meat represents a poor source of n-3 LC-PUFA; however, it is possible to increase their content by manipulating the broiler diet.

In this context, several studies [126,127] were conducted in order to evaluate the use of micro- and macroalgae in poultry nutrition as an n-3 LC-PUFA source. In particular, the use of Spirulina algae in the broiler diet increases the n-3 LC-PUFA content in meat, particularly EPA and DHA, with a positive effect also in the n6/n3 ratio [128]. The same results were obtained by Costa et al. [129] through the enrichment of the broiler diet with Brown macroalgae (e.g., *Laminaria digitata*). However, it is important to consider that both micro- and macroalgae are characterized by the presence of cell walls resistant to degradation by digestive enzymes. Thus, a high level of algae in an animal diet can compromise the nutrient digestibility, with a negative effect on the growth performance of animals [129].

Consequently, the main source of n-3 PUFA (ALA) used in the nutrition field is represented by flaxseed that is administered to the birds in different products, such as seeds, oil, or extract. Feeding turkeys with a diet containing 2.5% flaxseed oil from 16 days to 3 weeks of age before slaughter resulted in the recommended n-6/n-3 polyunsaturated fatty acids ratio of 4:1 in the meat [130].

Chickens, through the hepatic elongase and desaturase enzymes, are able to produce n-3 LC-PUFA from ALA [131]. Several studies demonstrated that it is possible to increase the level of n-3 LC-PUFA in chicken meat through the dietary supplementation of ALA. Other researchers did not obtain the same results, suggesting that there are other factors affecting the n-3 LC-PUFA biosynthesis. Diets with 8% of ALA increased the level of n-3 LC-PUFA in the chicken meat nine times as compared to the control group [132]. In contrast, Lopez-Ferrera et al. [133] reported that administering 8% of ALA in the diet obtained only a 3.6 times increase in n-3 LPC in the breast meat. The discrepancies found in the different studies are potentially the result of different durations of feeding ALA, the LA level in the diet, and genetic strain of birds in the studies (see Section 7 and Section 8).

Furthermore, the ALA sources can affect the organoleptic properties of chicken meat; for example, 10% flaxseed addition in the last 14 days of the rearing cycle did not affect its taste or aroma [134], while 7% of flaxseed oil addition, in the same period, produced a fishy odor and taste in chicken meat [135].

As previously affirmed, when the level of PUFA increases, a higher antioxidant protection should be obtained. Vitamin E is the main antioxidant used for fatty acids supplementation and is generally added as α-tocopheryl acetate in poultry diets. Vitamin E supplementation in broiler diets not only increases the total tocopherols concentration in the different tissues (liver > adipose tissue > dark meat > white meat) but also enhances the antioxidant defense of major tissues, decreasing lipid peroxidation [136].

A body of literature also underlines that, in poultry, the effects of the dietary treatments are tissue-specific [132]. In other words, is not possible to establish a linear correlation among the ALA level provided with the diet and the concentration of n-3 LC-PUFA found in the different muscles (mainly breast and drumstick) in poultry meat.

Table 3 highlights some data from the literature. Although the studies were carried out in different experimental conditions, the same trend of n-3 LC-PUFA distribution in relation to the tissues was observed. In particular, when chickens are fed with ALA and EPA supplementation, a higher level of n-3 LC-PUFA was found in the liver compared to the breast [137] and drumstick [138]. Moreover, the drumstick data show a higher ALA content in respect to the breast; the latter exhibits a higher concentration of n-3 LC-PUFA (EPA and DHA) [139,140]. This is probably due to the different roles performed by the breast and drumstick muscle. It is well-known that the drumstick is involved in the movement and such activity consumes energy obtained through two main sources: carbohydrates and free fatty acids. The carbohydrates (mainly glycogen) are used for a fast and short contracting activity, whereas the free fatty acids are involved in the slow and prolonged exercise [141]. For this reason, the drumstick is rich in fats compared to the breast, and a portion is used (β-oxidation) for kinetic activity [142].

In order to obtain meat rich in n-3 LC-PUFA, it is fundamental to consider both birds’ ability to convert ALA into n-3 LC-PUFA and their storage efficiency of such compounds in the edible tissues [143].

#### 5.1.2. Dietary Enrichment for Laying Hens

In poultry, as well as in other species [144,145], it has been demonstrated that the conversion of ALA is higher in females than in males [146]. As previously mentioned, sex has a significant effect on FADS expression and consequently on the n-3 LC-PUFA content of poultry products; adult female chickens have higher n-3 LC-PUFA synthesis ability due to needs of the chicken embryo. This is the reason why the efficiency of conversion of ALA into LC-PUFA varies between chicken meat and eggs. Accordingly, egg yolk is a good source of n-3 LC-PUFA, especially DHA; indeed, standard egg yolks contain 0.1% EPA, 0.7% DHA, and 0.8% ALA [147]. Over the past 20 years, the influence of the dietary supplementation (mainly flaxseed) on the productive performance of the hens and the characteristics of the eggs have been extensively studied; however, the results reported are highly variable. Several authors reported a decrease in feed consumption [148,149], while others an increase [150] when flaxseed was added to the diet. Moreover, concerning the egg production, some authors showed an increase [151], while others reported a decrease [148] or no change of deposition rate [152]. Similar discordance was observed for egg weight [148,152]. These contradictory results can be ascribed to differences in experimental conditions; in fact, it is known that the age of hens and the genotype influence productive performance [153,154]. However, in these studies, the most important factor is represented by the diet formulation and, in particular, by mechanical/chemical treatments of raw materials. Flaxseed and other seeds used as ALA source often contain antinutritional factors (ANFs) that negatively affect the palatability and the digestion efficiency of the diet [118,155]. The cyanogenic glycosides present in the flaxseed are ANFs, responsible for impaired respiration rate in laying hens. Moreover, other ANFs, such as phytic acid and trypsin inhibitors, increased the intestinal viscosity [156] by reducing nutrient bioavailability [157]. These effects reduce hen performance and affect the egg quality [158]. However, by mechanical processing (extruding, heating, or by enzyme supplementation) of the raw seeds, it is possible to reduce/eliminate the ANFs [159,160]. Recent studies reported that the flaxseed extrusion [159], or the use of oils or soluble ingredients as ALA source, did not affect the productive performance in laying hens [118,161]. Therefore, in order to increase the n-3 LC-PUFA content in the eggs, it is important to use mechanically processed sources of ALA to avoid the effect of ANFs.

Regardless, laying diets rich in ALA, such as flaxseed, can increase the EPA and DHA content of their eggs [162,163]. Fraeye et al. [164] in their review concluded that dietary supplementation of flaxseed in laying hens proportionally increases the level of ALA in the yolk. Furthermore, amounts of DHA in yolk increase as well, but not in a linear way with respect to the level of flaxseed supplementation, suggesting that the LA/ALA ratio of the diet is one of the major factors affecting LC-PUFA synthesis. As previously mentioned, the common enzymatic pathway between n-3 and n-6 PUFA induces a competition for their synthesis; thus, the level of ALA and LA in the diet represents a crucial aspect. A recent meta-analysis [165] showed a linear relationship between the ALA levels in the diet and the amount of EPA, DHA, and total n-3 LC-PUFA in egg yolks, whereas a decrease in LA concentration was simultaneously observed. Authors reported that adding 100 g of ALA per kg in the diet resulted in 126 mg of DHA in the egg. Additionally, it was confirmed that, by increasing the LA content in the diet, and, consequently, the LA/ALA ratio, there was a linear decrease in the concentration of EPA and DHA in egg yolks. Given these relationships, diet composition (concentration of ALA, n-3 PUFA, and LA/ALA) can predict the content of EPA and DHA in the egg with a certain accuracy. However, it is important to consider that, even though numerous studies show that the decrease in LA and the increase of ALA in animal feed promotes the synthesis of DHA [166,167], other researchers reported that the continuous exposure to diets characterized by a high gap of LA to ALA decreased the egg weight [168]. Dong et al. [169] affirmed that hens fed fish oil supplementation over 16 weeks resulted in decreased egg weight. Thus, it is necessary to maintain a balanced LA/ALA ratio in the diet. Additionally, the age of hen is important and affects the n-3 LC-PUFA synthesis. Older hens characterized by higher liver dimensions are more efficient in metabolizing DHA from ALA compared to younger hens [164].

Flaxseed addition also affects other nutritional traits of egg. Mattioli et al. [117] show that the supplementation of the hen’s diet with flax and alfalfa sprouts reduces plasma and egg cholesterol and increases the n-3 PUFA, vitamins (α-tocopherol, α-γ-tocotrienol, retinol), carotenes (β-carotene, lutein, zeaxanthin) and phytoestrogens (daidzein, equol, isolariciresinol).

It is important to underline that direct dietary alterations represent the main method for modifying LC-PUFA content in animal products; however, genetic [170] and rearing strategies [171] should not be ignored (see Section 6 and Section 7).

#### 5.1.3. Use of Insect and Earthworms as a Future Prospective

In recent years, many efforts have been made to find alternative and sustainable sources of feed for animals. One of the most promising sources is insects. Insects constitute more than 75% of the animal kingdom [172] and have potential as a sustainable source of food and feed. Most edible insects are rich in protein, lipids, and minerals. Insects and other invertebrates are natural protein sources for poultry and can potentially replace fish and soybean meal. Insects, due to their high reproductive potential, chemical characteristics, low water and space requirements, ability to use waste as feed, and low environmental impact, can be produced sustainably for livestock feed [173].

To increase sustainability, the diet for insects should consist of previously unused biomass, such as waste, low-value by-products, and non-traditional livestock feedstuffs.

However, currently, there are limitations on the types of feed allowed in insect rearing. Since insects are considered livestock, the ingredients allowed in insects’ diets are subjected to EU regulations on feed hygiene, which restricts the use of food sources such as catering waste and processed animal protein [174]. In addition, the nutrient composition of insects varies with species, age, life stage, production, and processing conditions [175,176,177]. Even though significant variations exist in the nutrient composition of edible insects, many of these insects are high in monounsaturated and/or PUFA, mainly LA, ALA, and γ-linolenic acid [178], whereas LC-PUFA are scarce. The fatty acid composition of insects also depends on the environment where they develop [178]. Terrestrial species have lower LC-PUFA content, especially lower n-3 LC-PUFA, compared to species with an aquatic larval stage [179] due to the major differences in n-3 LC-PUFA availability in terrestrial plants versus aquatic primary producers such as algae.

Theoretically, insects could contribute to n-3 PUFA and LC-PUFA requirements for humans either by direct consumption of insects rich in n-3 PUFA or indirectly through consumption of fish and poultry products fed on such insects [180]. Among edible insects, black soldier flies (BSF, *Hermetia illucens*) are one of the most-studied, easily reared, and widely approved insects for use in poultry feeds in the US and Europe. BSF larvae can be raised on a wide range of substrates, resulting in insect biomass suitable for feeding animals [181]. The FA composition of insects such as BSF is partly determined by the composition of their diet, which can be modified to achieve a favorable n-6/n-3 ratio for animal feed. However, Hoc et al. [182], investigating the long-chain metabolic activity of BSF larvae, found that such insects produce scarcely any LC-PUFA. For example, even when fed high levels of ALA from flax-enriched diets, the larvae bioaccumulated around 13% of this fatty acid and metabolized approximately two-thirds of it into saturated fatty acid, such as lauric or myristic acid.

In a recent study, Rossi et al., [183] demonstrated an LC-PUFA enrichment in yellow mealworm (*Tenebrio molitor*) larvae only when they were reared on diets enriched in fish oil but not on sunflower or flaxseed oil diets. Such results suggest that these insects (i.e., *T. molitor* or BSF) are not able to convert ALA into n-3 LC-PUFA, and, thus, they are not a potential source of these compounds for consumption. According with the above-reported studies, both BSF and *Tenebrio molitor* showed a scarce efficiency to convert ALA into n-3 LC-PUFA. Therefore, they are mainly used as a source of protein [184,185] and lipids [186,187] as replacements of conventional ones.

However, studies on other insects, such as mealworms, have demonstrated that adding a source of n-3 fatty acids to their diet can significantly increase the n-3 PUFA content of some insect meals [183,188,189]. For instance, a recent study showed that an inclusion of 4% flax seed oil in diet resulted in 10- to 20-fold increase in n-3 fatty acid content in house crickets, lesser in mealworms and BSF [190]. Other studies also indicated an increase in the n-3 LC-PUFA content in BSF larvae when raised on oil seed by-products, fish offals, or seaweed-based mediums [181,191,192]. Grass-fed poultry can naturally ingest various amounts of insects and earthworms. Earthworms are yet another alternative and little-explored source of protein and LC-PUFA for domestic animals. The use of earthworms presents a unique opportunity as earthworms can efficiently recycle the organic wastes and by-products from livestock operations into valuable feed sources for animals. Earthworms are high in essential amino acids and n-3 PUFA compared to other insects, and they can obtain EPA from their gut microflora instead of depending on dietary sources [193,194]. Earthworms are already part of a chicken’s natural diet and their effect on inclusion in poultry diets has been reported occasionally from various geographical regions across Asia [195,196,197,198,199,200]. Dietary supplementation of earthworm meal (0.2–0.6%) improved performance in broilers as well as layers, especially in terms of laying performance, egg quality, and n-6/n-3 ratio of FA in egg yolks [198]. The content of n-3 FA, mainly EPA and DHA, is also important in the earthworm flour. For instance, the flour of *E. Andrei* is recommended as a source of protein and lipid in fish feed because of its high protein and PUFA content [201]. The current regulations in Western countries (EU and USA) do not allow the use of earthworms as an animal feed when reared on wastes. For example, the current EU legislation only permits certain insect species, including BSF, common house fly (*Musca domestica*), the Coleopetran species yellow mealworm, lesser mealworm *(Alphitobius diaperinus*), house cricket (*Acheta domesticus*), banded cricket (*Gryllodes sigillatus*), and field cricket (*Gryllus spp.),* which have been reared on materials of vegetal origin in aquaculture feed, but prohibits raising insects or earthworms on catering or manure waste due to the risk of pathogen transmission [202]. However, there are fewer restrictions in Asia, Africa, and America in terms of insect species and rearing substrates.

More research is needed to identify the best substrates to raise earthworms, the safety of the feed sources, and the identification of the best species of earthworms to be reared for use in poultry and aquaculture.

## 6. Poultry Genotype

The current European regulations do not provide a clear classification of meat-type chicken genotypes, so most EU countries make this discrimination on the basis of daily weight gain (DWG). Therefore, broilers can be divided into three major groups based on their productivity:

1. Fast-growing genotypes (FG) are represented by birds used in intensive rearing systems reaching commercial weight in a very short time and characterized by a high breast yield (> 25% live weight). The most common genotypes are selected for precocity; at about 40 days of age their weight is more than 2.5 kg;
2. Medium-growing genotypes (MG), also known as slower-growing genotypes (SrG), are a recently recognized group and comprise some commercial chicken genotypes that are lower-performing then the FG ones, which is why the breed companies define them as SrG. These genotypes are less common in intensive rearing systems, but they are widely used in alternative rearing systems (e.g., free-range and organic);
3. Slow-growing genotypes (SG) are represented by breeds that are very important for maintaining biodiversity and genetic variability but, due to their low productive performance (growth rate and breast yield), are not utilized in intensive rearing systems. Thus, they are mainly used for niche-production in small-scale farms [203].

In the alternative systems, the presence of outdoor runs renders necessary the use of suitable genotypes with specific features, such as: kinetic and foraging activity, thermo-tolerance, and immune response of the organism [204,205]. It is widely known that FG genotypes use most of their dietary energy towards body growth, while the SG genotypes spend most of their energy on metabolic functions, such as thermoregulation, movement, and foraging [206]. Sirri et al. [207], comparing organically reared SG, MG, and FG chickens, found higher n-6 and n-3 PUFA concentration in the breast of SG, suggesting differential expression of genes encoding for desaturating enzymes. However, since SG and MG birds are reported to eat more grass than FG genotypes [208], their higher dietary ALA intake could have also contributed to the higher degree of unsaturation in their meat.

Additional papers [209] confirmed that MG, and particularly SG chickens, showed a greater expression of FADS2 and FADS1 genes and a higher Δ6 and Δ5 activity and, consequently, higher n-3 LC-PUFA content in breast meat compared to FG genotypes. These findings agree with resource allocation theory [206] because the synthesis of n-3 LC-PUFA, due to additional cycles of elongation and desaturation (Figure 2 Section 3) and a final β-oxidation, is more metabolically expensive than that of the n-6. This could explain the preference of the FG, compared to SG, for n-6 [210]. As previously discussed, the competition between the two metabolic pathways (n-3 and n-6) affects the relative synthesis of different LC-PUFA. Dal Bosco et al. [211] show that the estimated Δ^5^/Δ^6^-desaturase index was higher in the SG as compared to the FG genotypes and, consequently, the SG meat was characterized by a higher percentage of LC-PUFA (both n-3 and n-6) with respect to the FG meat. Accordingly, genetic selection for high performance unintentionally modified the expression of genes coding for enzymes involved in LC-PUFA synthesis as well as the relative enzymatic activity [212]. The relationship between genotype and desaturating ability has been demonstrated to have a significant impact on the PUFAs content of meat.

A recent paper [143] underlines that the presence of LC-PUFA in chicken meat depends on two main factors: the liver desaturase activity of the bird (conversion of ALA into LC-PUFA) and the storage capability of the LC-PUFA synthesized in tissues (mainly muscle). This study confirms the higher desaturase ability of the SG compared to the FG genotype but also points out the higher muscle storage of the FG chickens with respect to the SG as a consequence of their higher body fat content. SG birds more efficiently synthesize LC-PUFA, leading to a higher percentage of n-3 and n-6, but FG birds have a higher storage capability and, consequently, higher muscle fatty acids concentrations (in terms of mg fatty/100 g tissue). Overall, the research suggests that genetic effects on the desaturase and elongase activities are responsible for variation in n-3 LC-PUFA synthesis, making the genetic mechanisms behind the synthesis very interesting for future research. A new challenge in genetic selection should be to find a genotype with an optimum equilibrium between LC-PUFA synthesis and storage ability in order to increase the LC-PUFA content in chicken products. The identification of this genotype might represent an important goal not only for the agro-industry but also for the improvement of human nutrition.

For laying hens, genetic effects are less studied and probably less relevant because laying hens maintain high LC-PUFA conversion efficiency, mainly modulated by the reproductive efficiency (e.g., deposition rate) of their genetic strain [171].

## 7. Rearing System

The EU produces about 13.4 million tons of poultry meat [213], and 95% of this meat comes from intensive rearing systems [214]. These data show that poultry meat production from alternative systems (e.g., organic and free-range) is very limited. However, in recent years, there has been increasing demand for poultry meat and eggs grown utilizing these systems. In this context, France dominates the EU market by producing 16% of the total chicken meat with outdoor rearing systems [215].

**Figure 5:**
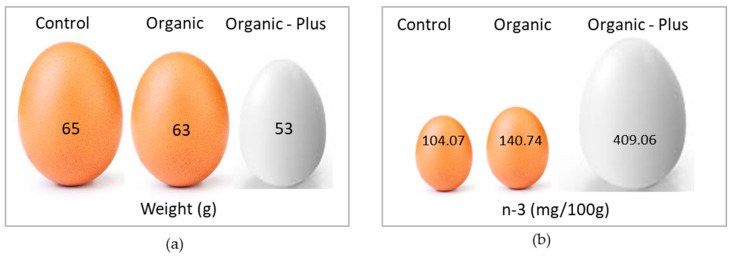
Comparison between (**a**) the weight (g) and (**b**) n-3 content (mg/100 g) of eggs from hens reared in conventional, organic, and organic–plus system. Conventional rearing system was characterized by commercial hens reared indoors; in the organic rearing system, commercial hens with 4 m^2^/hen of organic pasture were used, whereas organic-plus system consisted of local breed hens with 10 m^2^/hen of organic pasture. Data from Dal Bosco, 2016 [216].

In alternative poultry production, the presence of a pasture is crucial since the foraging birds spend a lot of time outdoors eating forage, pebbles, weeds, crop seeds, earthworms, and insects [204,216,217]. Many studies [171,217,218] have assessed the effects of pastures on poultry meat and egg quality. A natural pasture is rich in n-3 precursors, vitamins, and antioxidants that are transferred to chicken products, thus improving the fatty acid profile and the oxidative status of the meat and eggs with respect to those obtained from animals fed only commercial feedstuffs [216,219]. For example, the presence of a pasture reduced the n-6/n-3 ratio in SG chickens compared to the same strains when conventionally reared [220]. Dal Bosco et al. [216] also found a higher antioxidant intake in chickens reared outdoors compared to those reared indoors. Consequently, the antioxidant capacity of the plasma and the antioxidant levels of the meat were also greater in the outdoor groups than in the indoor ones. Hens with access to a pasture produce eggs with at least twice as much vitamin A and E and n-3 LC-PUFA compared to hens with no access to a pasture [221]. The forage intake of hens also positively influences the FA profile of egg yolks. Organic-plus hens (local breed with 10 m^2^/hen of organic pasture) showed eggs with higher content of n-3 PUFA and lower concentrations of n-6 PUFA as compared to organic (commercial hens with 4 m^2^/hen of organic pasture) and conventional (commercial hens indoor-reared) eggs (Figure 5, [216]). Moreover, SG chickens reared outdoors are able to express all of their behavioral repertoire, mainly characterized by the foraging activity that naturally enriches their products with LC-PUFA and antioxidants [205].

When alternative rearing systems are adopted as a strategy to increase the LC-PUFA in animal products, it is important to choose suitable genotypes for this type of rearing (Section 6). It is widely recognized that FG genotypes, selected for high productive performance, being static animals, reared in outdoor conditions, exhibit low grazing behavior without exploiting the beneficial effects of a pasture [204,214].

## 8. Animals Transport

As discussed previously, the nutritional quality of poultry meat is affected by many factors, such as diet, genetic strain, rearing system, and animal welfare during the rearing phase. Other crucial factors that affect meat quality include the transport conditions of poultry from the farm to the slaughterhouse [222].

Chickens are captured and then, during transport, they are caged and deprived of water and feed and can be subjected to variable environmental conditions (i.e., noise, vibrations, and differences in temperature and humidity). European rules set several parameters for the humane transfer of poultry to the slaughterhouse; however, birds may still be subjected to high stress. Authors [223] show that chickens transported for 4h (which could be considered an average transport time in commercial conditions) exhibit higher stress (determined by a higher Heterophils/Lymphocytes ratio, an indicator of stress) compared to non-transported chickens. Research suggests that the effect of stress could be different in FG and SG strains [224]. Berri et al. [225] reported that SG suffer more during the lag phase between catching and slaughter due to their high kinetic activity (i.e., wing flapping) during transport and slaughtering. Accordingly, SG chickens, being more active, seem more sensitive to stress during transport compared to FG [226]. Moreover, the higher energy expenditure due to the metabolism increase caused by the stress consumes PUFA via β-oxidation, reducing the concentration in body tissues [142]. Notably, the length of transport increases the proportion of saturated fatty acids of breast meat (mainly C16:0 and C18:0) and decreases PUFA (LA, AA, EPA, and DHA) content, likely due to greater formation of peroxides, as confirmed by higher TBARS values.

In addition, oxidative stress, caused by the length of transport, reduces the in vivo antioxidant content of the body and, consequently, enhances the post-mortem susceptibility of PUFA to lipid oxidation. Cartoni Mancinelli et al. [227], through the mobile poultry processing unit (MPPU), have proposed a possible solution applicable to small-scale farmers to avoid/reduce the transport of the birds. The MPPU, the first in Europe, consists of a truck equipped with a small slaughterhouse able to reach the poultry farm. At the moment, positive conclusions exist concerning the effect of MPPUs on animal welfare and meat quality.

Thus, to improve and preserve the nutritional quality of poultry meat, it is very important to pay particular attention both during the rearing period of animals (genotypes, breeding systems, diet, animal welfare, and health) and in the successive phases, such as slaughtering and cooking procedures.

## 9. Cooking Procedures

Meat is a perishable food; it has been reported that lipid oxidation of meat occurs in the following order: fish > poultry > pork > beef > lamb [228]. This different susceptibility to oxidation is attributed to the level of unsaturated fatty acids, as ALA oxidation is 20 to 30 times higher than LA [229], and to the antioxidant availability, in particular vitamin E stored in muscle cells. The cooking temperature of meat can result in the development of volatile organic compounds (VOC), which is largely attributed to the autoxidation of PUFA. The secondary products of lipid oxidation are responsible for warmed-over flavor production (WOF) [230]. The organoleptic rancidity due to the oxidative deterioration negatively affects acceptability for consumers. Pearson et al. [231] show that the temperature of 70 to 80 °C damages muscle membranes, which, in turn, induces the interaction of lipid oxidation catalysts with unsaturated fatty acids and consequent production of free radicals.

Among the free radicals, the thioradicals (e.g., oxidized proteins) are largely responsible for the WOF [232]. Poultry meat, due to its high content of LC-PUFA, is very sensitive to the oxidative process and, as previously reported, the genotype represents the main factor affecting the concentration of PUFA in meat. Indeed, it has been widely demonstrated that the SG chicken genotypes are more efficient at synthesizing n-3 LC-PUFA as compared to the FG strains. A recent study [233] compared the fatty acid, antioxidants, and VOC content of raw and cooked meat samples derived from four chicken genotypes with different growth rates. The study showed a 5.5 times increase in VOC production in cooked meat compared to raw meat across genotypes. However, the VOC production level was related to the LC-PUFA content in the raw meat and to the genotype. Consequently, the SG meat was considered “more vulnerable” due to the lower content of antioxidants as compared with the FG genotype [233]. In fact, the SG birds, due to their innate higher kinetic activity with respect to FG, consume the dietary antioxidants also to balance the oxidative process induced by movement [234]. In order to contain the losses of n-3 LC-PUFA, the meat antioxidants/LC-PUFA ratio should be considered as these could be altered by the cooking process.

## 10. Conclusions

The health importance of n-3 LC-PUFA in humans, together with the inefficiency of n-3 LC-PUFA synthesis, has increased interest in enriching foods in these compounds. Poultry can be considered a suitable animal model for studying n-3 LC-PUFA enrichment strategies in terms of rearing cycle practices and post-mortem processes. The main method of dietary manipulation consists of supplementation of n-3 PUFA precursors and vitamin E without compromising the diet LA/ALA balance. The genotype choices also have an important role in determining the poultry n-3 LC-PUFA content since it is necessary to consider both the bird’s ability to convert ALA into n-3 LC-PUFA and the deposition of such compounds into the edible tissues of chickens. When alternative rearing systems are used as a strategy to increase the n-3 LC-PUFA in animal products, due to the presence of ALA in grass and insects, it is crucial to adopt an explorative genotype able to use the available pasture. In order to preserve the n-3 LC-PUFA accumulated by the chickens during the rearing cycle in poultry products, it is also important to pay attention to the next steps, such as animal transport to the slaughterhouse and cooking procedures. For both processes, it is essential to reduce the duration and ensure an adequate quantity of dietary antioxidants in order to preserve the products’ quality. For enriching and preserving the n-3 LC-PUFA in poultry products, a multifactorial approach should be adopted that encourages the use of multiple strategies throughout the entire production chain.

Further research efforts are still needed to clearly define the storage efficiency of the different strategies for the enrichment of poultry meat and eggs. Certainly, in the current world context, with insufficient n-3 LC-PUFA supplies for human nutrition, it is necessary to apply responsible and sustainable approaches, including:

1. avoiding livestock and human competition for n-3 LC-PUFA;
2. developing livestock systems with the best conditions for bio-conversion of n-3 precursors into n-3 LC-PUFA.

## Figures and Tables

**Table 1:** Estimated global requirements of n-3 LC-PUFA and their availability from fish.

n-3 LC-PUFA REQUIREMENT			
Population classes	n	EPA and DHA (mg/d)	EPA and DHA (t/y)
Total world population	7,922,857,397		
Adult individuals	7,903,361,753	250	356
Women in pregnancy and lactation	9747,822	400	1423
Young children (<24 months)	9,747,822	100	721,182
TOTAL			~722,960
AVAILABILITY			
Wild and farm-raised fish			100,000,000
50% of fish is suitable for human consumption			50,000,000
15% of the n-3 represent EPA and DHA			375,000
DEFICIT			~347,956

The table shows the estimation of the global annual deficit of n-3 LC-PUFA (t/y) calculated considering the n-3 LC-PUFA requirement in the main population classes and the amount of EPA and DHA provided by fish.

**Table 2:** Estimated global availability of n-3 LC-PUFA from terrestrial sources [110,111]).

	t/y	mg LC-PUFA/d	t LCP/y
Poultry	1.3 × 10^8^	0.62	40,300
Eggs	7.7 × 10^7^	0.35	26,845
Pork	9.4 × 10^7^	0.18	8487
Total			75,632

The table shows the estimated annual availability of the n-3 LC-PUFA (mg/d or t/y) obtained from the monogastric livestock mostly consumed in the human diet.

**Table 3:** n-3 fatty acid profile (mg/100 g of fresh tissue) in liver, breast, and drumstick tissues of broilers fed with different ALA and EPA sources.

Tissues	ALA+EPA g/kg of Diet	Time Feeding (d)	Genotype	ALA (C18:3)	EPA (C20:5)	DHA (C22:6)	TOTAL	References
Breast	15	44	Ross 308	18.0	3	10	31	Cortinas, 2004 [139]
Drumstick				197	7	17	221	
Breast	25	21	Ross 308	147	13.5	31.5	192.0	Rymer, 2006 [140]
Drumstick				258	10.8	17.5	286.3	
Liver	6.1	6	Cobb × Ross 308	67.1	280.0	120.1	467.2	Shin, 2012 [138]
Breast				52.3	36.1	20.4	108.8	
Drumstick				104.7	38.8	15.6	159.1	
Breast	10	30	Ross 308	185	56	86	327	González-Ortiz, 2013 [137]
Liver				407	275	335	1017	

The table shows higher level of n-3 LC-PUFA in the liver compared to the other tissues. The drumstick exhibits a higher ALA content in respect to the breast.

## Data Availability

Not applicable.
